# Development and validation of a novel anoikis-related gene signature in clear cell renal cell carcinoma

**DOI:** 10.3389/fonc.2023.1211103

**Published:** 2023-10-26

**Authors:** Qihang Wu, Yuxiang Sun, Xiangcheng Qin, Maomao Li, Shuaishuai Huang, Xue Wang, Guobin Weng

**Affiliations:** ^1^ Health Science Center, Ningbo University, Ningbo, Zhejiang, China; ^2^ Department of Emergency, Ningbo Yinzhou No.2 Hospital, Ningbo, Zhejiang, China; ^3^ Department of Urology, Ningbo Yinzhou No.2 Hospital, Ningbo, Zhejiang, China; ^4^ Urology and Nephrology Institute of Ningbo University, Ningbo Yinzhou No.2 Hospital, Ningbo, Zhejiang, China

**Keywords:** clear cell renal cell carcinoma, anoikis, signature, prognosis, immune infiltration

## Abstract

**Background:**

Despite numerous treatments available, clear cell renal cell carcinoma (ccRCC) remains a deadly and invasive cancer. Anoikis-related genes (ARGs) are essential regulators of tumor metastasis and development. However, the potential roles of ARGs in ccRCC remain unclear.

**Methods:**

Based on the TCGA-KIRC cohort and GeneCards database, we identified differentially expressed ARGs in ccRCC. Then a 4 ARGs risk model was created by Cox regression and LASSO. The Kaplan-Meier and receiver operating characteristic (ROC) curves were utilized to verify the predictive efficacy of the prognostic signature. Subsequently, the possible molecular mechanism of ARGs was investigated by functional enrichment analysis. To assess the immune infiltration, immune checkpoint genes, and immune function in various risk groups, single sample gene set enrichment (ssGSEA) algorithm was employed. Furthermore, the low-risk and high-risk groups were compared in terms of tumor mutation burden (TMB). Ultimately, we analyzed the protein expression of these four ARGs utilizing the western blot test.

**Results:**

Four genes were utilized to create a risk signature that may predict prognosis, enabling the classification of KIRC patients into groups with low or high risk. The reliability of the signature was examined utilizing survival analysis and ROC analysis. According to the multivariate Cox regression result, the risk score was a reliable independent prognostic predictor for KIRC patients. The novel risk model could differentiate between KIRC patients with various clinical outcomes and represent KIRC’s specific immune status. An analysis of the correlation of TMB and risk score indicated a positive correlation between them, with high TMB being potentially linked to worse outcomes.

**Conclusion:**

Based on our findings, the prognostic signature of ARGs may be employed as an independent prognostic factor for ccRCC patients. It may introduce alternative perspectives on prognosis evaluation and serve as a prominent reference for personalized and precise therapy in KIRC.

## 1 Introduction

Renal cell carcinoma (RCC) is known as a prevalent and lethal disease, accounting for 431288 new cases and 179368 fatalities worldwide in 2020 (1). Clear cell RCC (ccRCC) is the most prevalent form of RCC and accounts for >70% of tumors that arise from the renal epithelium (2). Despite the many different therapeutic modalities available, including surgery, targeted therapy, chemotherapy, radiation, and the recently suggested immunotherapy, ccRCC remains one of the most challenging clinical issues in urology (3). Delays in diagnosis and a high metastatic rate are the leading causes. Patients with metastatic RCC still have an unfavorable prognosis, with a 5-year overall survival rate of< 15% (4). Thus, it is urgent to discover new potential biomarkers and predictive models for the clinical management of ccRCC.

The term anoikis describes a form of programmed cell death that happens when cells separate from their usual extracellular matrix, thereby interfering with integrin ligation (5). Anoikis is essential for preserving homeostasis of the tissue and facilitating the progress by inhibiting the colonization of detached epithelial cells in other regions (6). The scientific community has paid considerable attention to anoikis resistance as it is associated with critical phases of tumor progression, such as epithelial-mesenchymal transition and anchorage-independent growth, as well as the metastatic spread of cancer cells, primarily due to the disruption of anoikis regulation (7). Accumulating evidence indicates that anoikis-related genes (ARGs) are critical in regulating tumor metastasis and cancer progression. For example, overexpression of CPT1A is related to poor clinical results for esophageal squamous cell carcinoma, while disruption of CPT1A may prevent tumor metastasis and anoikis resistance (8). In addition, it was found that HER2 overexpressing breast cancer cells exhibited heightened resistance to anoikis, and the HER2/GSK3/GLI2 pathway was identified to have a new function in the resistance to anoikis and metastasis (9). A recent investigation found that TCF7L2 exhibits high expression levels in gastric cancer (GC) and is an independent risk factor for bad prognosis in GC patients, with evidence suggesting that it contributes to both anoikis resistance and the metastatic cascade (10). Moreover, in ccRCC, Tim-3 expression disruption may decrease RCC invasion by accelerating anoikis (11). While the connection between anoikis and prognosis has been established in multiple cancers, there have been few studies on an effective predictive model based on ARGs in RCC.

Herein, we conducted a comprehensive analysis of data from The Cancer Genome Atlas (TCGA) KIRC cohort to investigate the connection between ARGs and clinical outcomes in KIRC. A new gene signature associated with anoikis was generated and validated, acting as an independent prognostic marker in ccRCC. Furthermore, when used in conjunction with clinicopathological features, a nomogram was devised using the signature as a basis, demonstrating effective predictive capabilities for ccRCC. Moreover, the study employed immune infiltration and functional enrichment analyses to investigate the fundamental mechanisms. In our study, we were able to identify a potential ARGs-based signature and apply it clinically in KIRC patients.

## 2 Materials and methods

### 2.1 Data collection and preprocessing

The TCGA database (https://portal.gdc.cancer.gov/) was utilized to collect the transcriptomic profiling of RNA-seq data of KIRC patients. Our study included 72 normal and 541 KIRC samples. Subsequently, we gathered the corresponding clinical data for these patients from TCGA. Samples lacking corresponding clinical characteristics or possessing a survival time of less than zero were eliminated, resulting in the inclusion of 532 KIRC samples for the subsequent analysis in this research.

### 2.2 Collection of differentially expressed anoikis-related genes

From GeneCards, 496 ARGs were retrieved by relevance score > 0.4 (**Supplementary Table S1**). The study utilized differentiation analysis with the “limma” package in R software (log2|fold change (FC)| >1 and adjusted *P*< 0.05) to identify differentially expressed genes (DEGs) in KIRC, comparing all gene expressions in normal and tumor samples using the Wilcoxon test. Afterward, by interacting with ARGs, differentially expressed ARGs were gathered.

### 2.3 Identification of anoikis-related signature

The 532 patients with KIRC were separated into training (n = 266) and test (n = 266) groups at random. **Table 1** provides a breakdown of the demographic characteristics for the training, test, and entire sets. The training and test sets did not show any statistically significant differences in their clinicopathological variables

**Table 1:** Characteristics of training set, test set and entire set of ccRCC patients.

	Training set, N=266	Test set, N=266	Entire set, N=532	P
	Number (%)	Number (%)	Number (%)	
**Age**				0.465
≤65	170(63.91)	179(67.29)	349(65.6)	
>65	96(36.09)	87(32.71)	183(34.4)	
**Gender**				0.468
Female	98(36.84)	89(33.46)	187(35.15)	
Male	168(63.16)	177(66.54)	345(64.85)	
**Grade**				0.871
G1	6(2.26)	8(3.01)	14(2.63)	
G2	112(42.11)	116(43.61)	228(42.86)	
G3	106(39.85)	100(37.59)	206(38.72)	
G4	36(13.53)	40(15.04)	76(14.29)	
unknow	6(2.26)	2(0.75)	8(1.5)	
**Stage**				0.447
I	139(52.26)	127(47.74)	266 (50)	
II	24(9.02)	33(12.41)	57(10.71)	
III	58(21.8)	65(24.44)	123(23.12)	
IV	44(16.54)	39(14.66)	83(15.6)	
unknow	1(0.38)	2(0.75)	3(0.56)	
**T**				0.755
T1	141(53.01)	131(49.25)	272(51.13)	
T2	31(11.65)	38(14.29)	69(12.97)	
T3	89(33.46)	91(34.21)	180(33.83)	
T4	5(1.88)	6(2.26)	11(2.07)	
**M**				0.908
M0	207(77.82)	214(80.45)	421(79.14)	
M1	40(15.04)	39(14.66)	79(14.85)	
unknow	19(7.14)	13(4.89)	32(6.02)	
**N**				0.420
N0	123(46.24)	117(43.98)	240(45.11)	
N1	6(2.26)	10(3.76)	16(3.01)	
unknow	137(51.5)	139(52.26)	276(51.88)	

To create an anoikis-associated signature, we first identified potential predictive genes in the training set based on differentially expressed ARGs using the univariate regression method. A machine learning technique called LASSO was employed to reduce overfitting. Furthermore, multivariate Cox regression was employed to create a new anoikis-related signature. A risk score formula according to the signature we built was established as follows:


$$\text{Risk Score}=\sum_{i=1}^{n}Coef(i)\times \exp(i)$$

where *Coef (i)* and exp(*i*) denote the expression value and the regression coefficient of eachprognosis related anoikis gene for each patient. The study categorized the cases into high-risk and low-risk groups based on the threshold of the median risk score.

### 2.4 Establishment and validation of nomogram

We performed univariate and multivariate Cox regression analyses to examine the independence of the risk model through the “survival” package. Clinicopathological characteristics (age, gender, grade, stage) and risk score were employed to generate a nomogram using the “rms” package, and all variables were computed and examined to determine the survival probability of ccRCC patients at 1, 3, and 5 years. To assess the consistency of the predicted and actual survival outcomes, calibration curves were employed. Moreover, receiver operating characteristic (ROC) curves were employed to confirm the prognostic precision of the risk score and clinicopathologic factors via the “pROC” package.

### 2.5 Functional enrichment analysis

The “limma” package was employed to determine differentially expressed genes in the low-risk and high-risk groups. The analysis employed a cutoff of log2| FC | >1 and adjusted *P*< 0.05. Furthermore, the Kyoto Encyclopedia of Genes and Genomes (KEGG) and Gene Ontology (GO) enrichment analyses were performed using the “clusterProfiler” package.

### 2.6 Immunity analysis of the signature

Six enrichment analysis algorithms, including the TIMER, MCPcounter, xCell, CIBERSORT, QuanTIseq, and EPIC algorithms, were utilized to evaluate cellular elements or cellular immune responses. The “GSVA” package was utilized to determine the immune function scores for all cases. Additionally, we contrasted immune checkpoints expression levels of the low-risk and high-risk groups. In addition, we assessed the scores of tumor immune dysfunction and exclusion (TIDE) for each ccRCC sample by utilizing the TIDE database (http://tide.dfci.harvard.edu/login/) for predicting the possibility of an immunotherapeutic response.

### 2.7 Mutation analysis

We utilized the TCGA dataset to obtain the mutation data of KIRC patients. The data comprising somatic variations were then evaluated and summarized via the “maftools” package.

### 2.8 ccRCC sample collection and western blot assay

Samples of both ccRCC tissues and adjacent normal tissues were provided by the Department of Urology at Ningbo Yinzhou No.2 Hospital. It is necessary to respect the privacy of the individuals involved in the study. The Institutional Research Ethics Committee of Ningbo Yinzhou No.2 Hospital has approved the study (batch number: 2023-KY-003).

To conduct the western blot analysis, RIPA buffer (Solarbio) that contained a protease inhibitor (1% PMSF, Solarbio) was used to lyse the ccRCC and adjacent normal tissues. 30μg of protein lysates were then isolated utilizing 10% SDS-PAGE gels and shifted onto a Millipore PVDF 0.45µm membrane. After the membranes were placed in TBST buffer with 5% non-fat milk to block, specific primary antibodies were added and left to incubate overnight at a temperature of 4°C. In this research, the primary antibodies utilized were: KIF18A (rabbit polyclonal, 1:1000; ab72417, Abcam), BID (rabbit polyclonal, 1:1000; 10988-1-AP, Proteintech), CHEK2 (rabbit polyclonal, 1:1000; 13954-1-AP, Proteintech), CEACAM4 (rabbit polyclonal, 1:1000; A10055, ABclonal), and β-actin (rabbit polyclonal, 1:50,000; AC026, ABclonal). Following extensive washing with TBST buffer, the membranes were then subjected to secondary antibodies (goat anti-rabbit IgG, 1:5000, AS014, ABclonal) at room temperature for another hour. The presence of stains was identified using an advanced chemical reaction known as enhanced chemiluminescence (ECL) reagent from the company Beyotime. The resulting protein bands were then examined using a gel imaging system (Tanon, China).

### 2.9 Expression analysis of ARGs in the signature at mRNA and protein levels

TCGA and GTEx (https://gtexportal.org/) were utilized to obtain the mRNA expression levels of 541 tumor samples and 100 normal samples of ARGs in the signature. In addition, we accessed TCGA to acquire mRNA expression data for the signature ARGs across 72 paired ccRCC and adjacent normal tissue samples for paired analysis. Finally, five matched pairs of ccRCC and paracancer tissues from our center were used to evaluate protein levels of the signature ARGs.

### 2.10 Statistical analysis

To compare the differential functions of the two groups, we employed the Wilcoxon rank-sum test. The survival disparities of the two cohorts were evaluated utilizing the Kaplan-Meier survival curve analysis. The pairwise differences between groups were evaluated utilizing the Student’s t-test. All analyses were executed on the R software (version 4.1.3) and a statistical significance threshold of *P*< 0.05 was applied.

## 3 Results

### 3.1 Identification of differentially expressed ARGs

**Figure 1:**
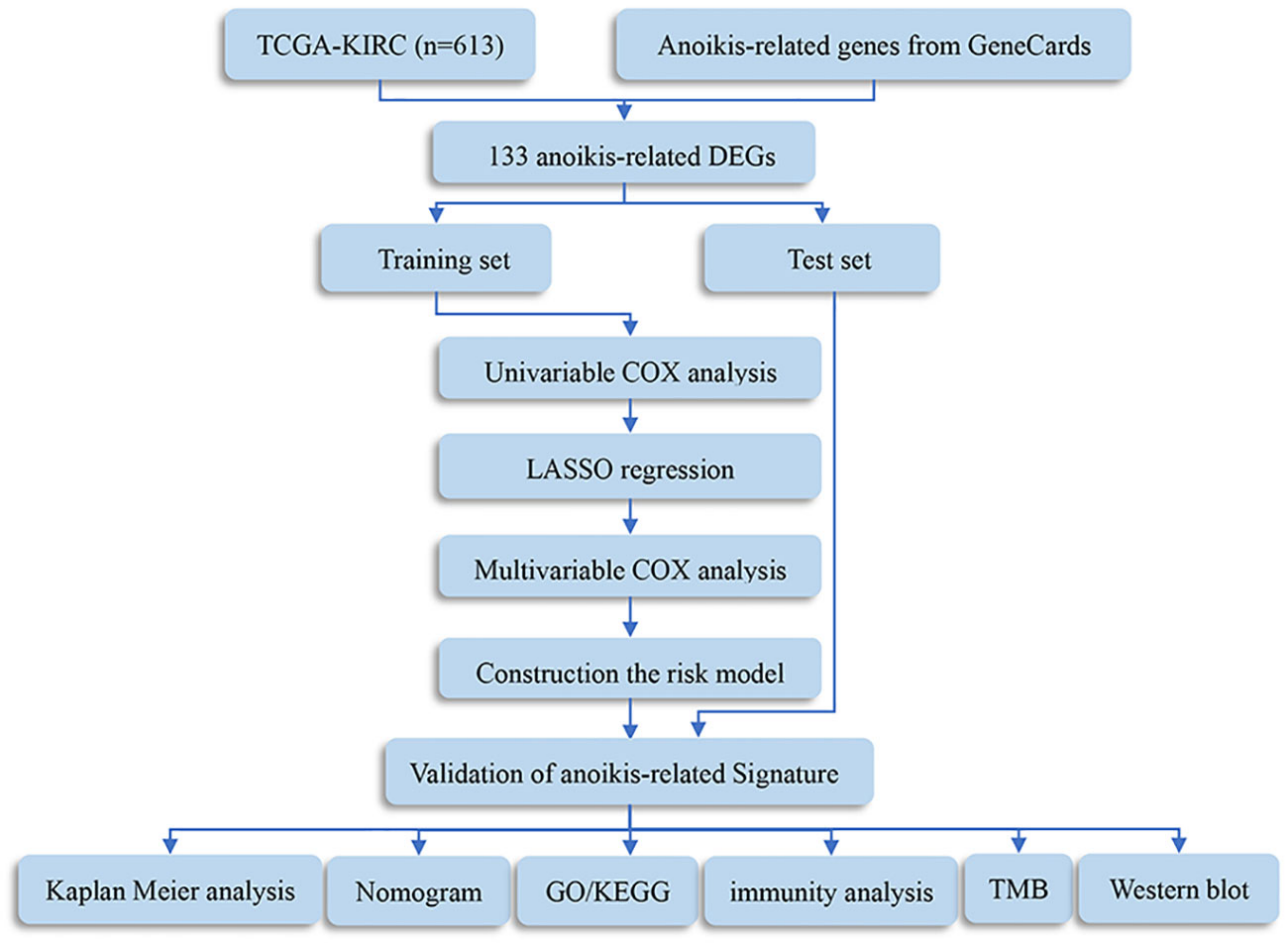
Flowchart of this study. DEGs, differentially expressed genes; TMB, tumor mutation burden; GO, Gene Ontology; KEGG, Kyoto Encyclopedia of Genes and Genomes.

**Figure 2:**
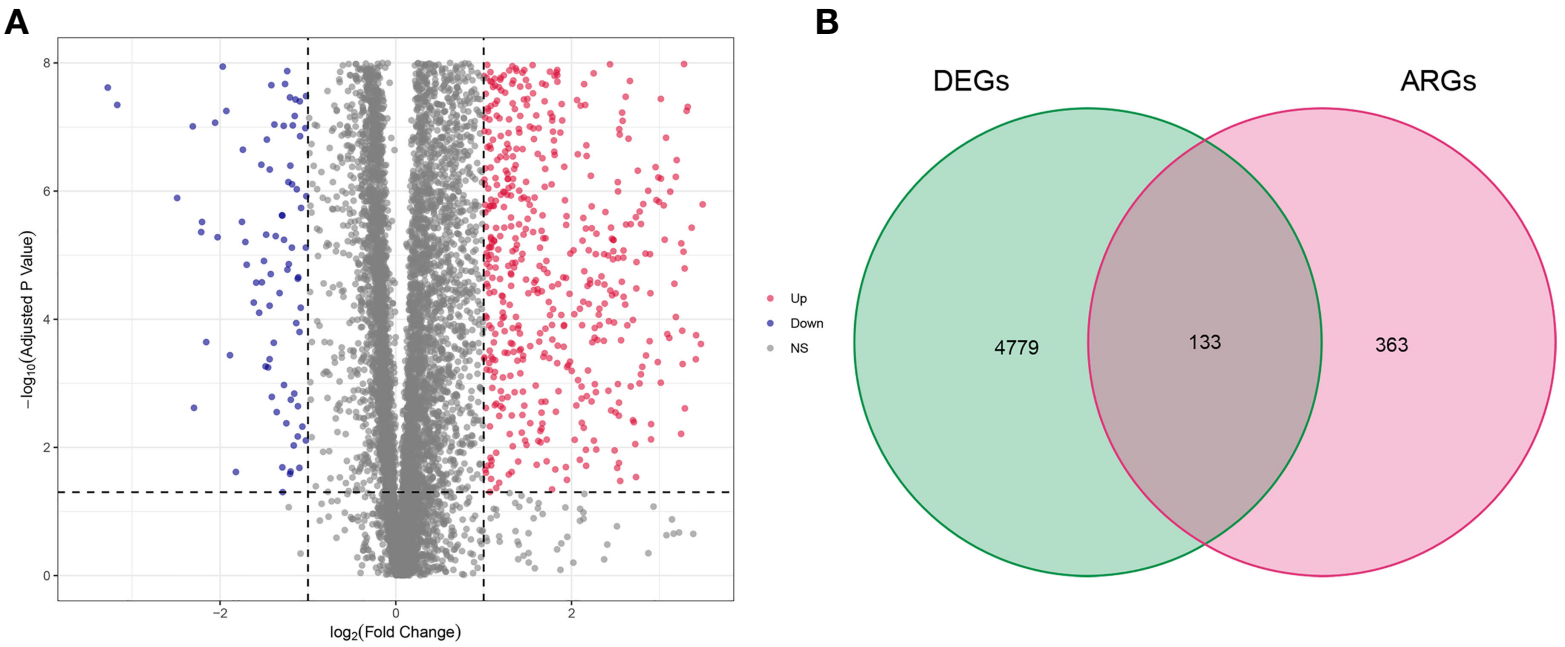
Result of differentially expressed anoikis-related genes (ARGs). **(A)** Volcano plot of differentially expressed genes (DEGs) in clear cell renal cell carcinoma (ccRCC). **(B)** Venn diagram showing the overlapping genes between DEGs and ARGs.

The procedure flow for our research is displayed in **Figure 1**. After evaluating data of gene expression from KIRC samples and normal controls in TCGA, we collected 4912 DEGs (**Figure 2A**). The set of DEGs was compared with 496 ARGs obtained from GeneCards. As a result of this comparison, 133 ARGs were determined as being both expressed differentially and shared between the two sets (**Figure 2B**).

### 3.2 Development of the ARGs prognostic signature

**Figure 3:**
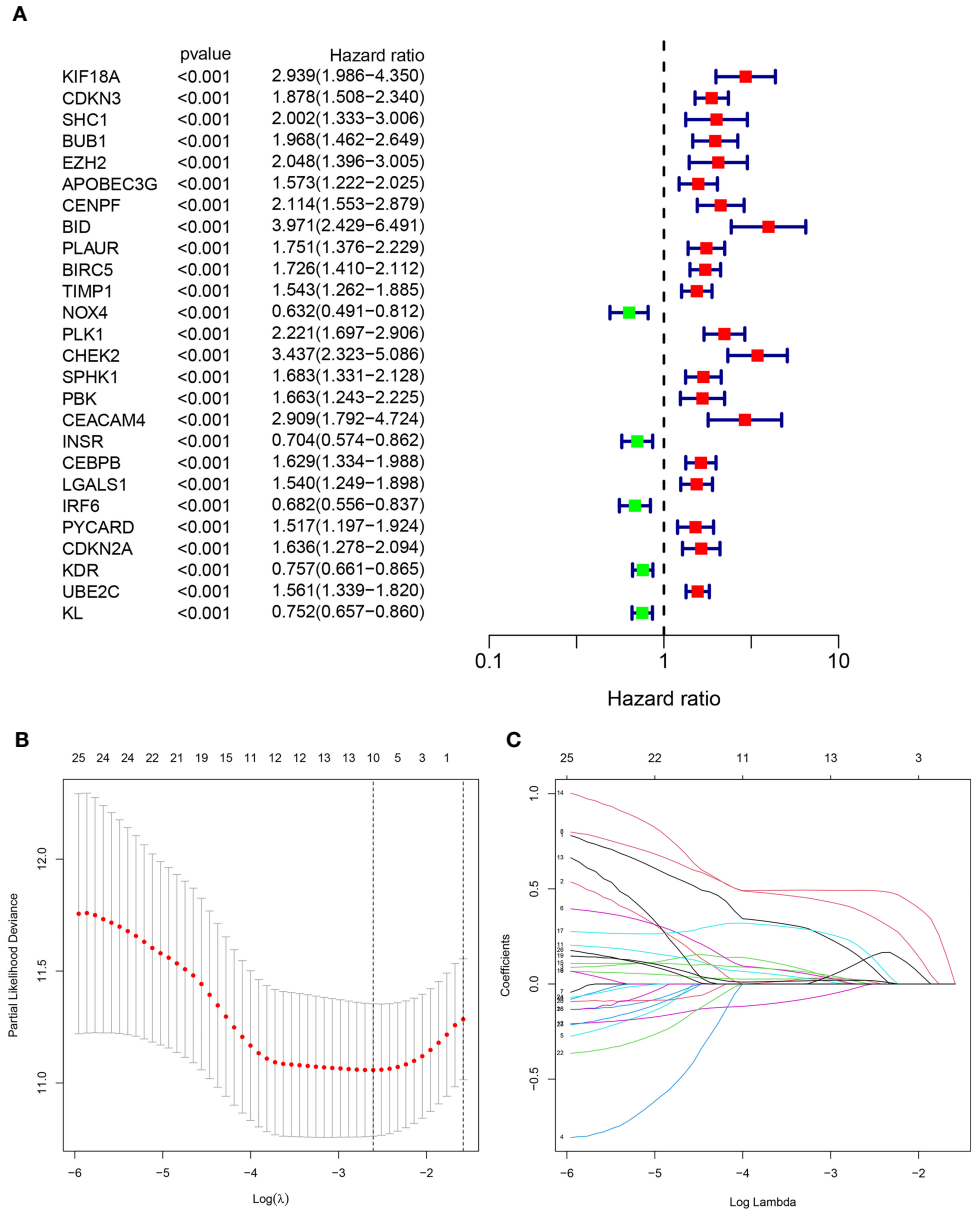
Identification of the anoikis-related signature (ARS). **(A)**The forest figure for Univariate Cox regression analysis of the ARGs. **(B, C)**. LASSO regression analysis shows the minimum lambda and optimal coefficients of the prognostic ARGs.

532 patients diagnosed with KIRC were assigned randomly into two groups: a test set (n=266) and a training set (n=266), as presented in **Table 1**. We employed univariate Cox method to identify 26 significant predictive ARGs from the training set (**Figure 3A**). We then performed LASSO-penalized regression to exclude the overfit gene of signature (**Figures 3B, C**). Finally, the signature of four ARGs (KIF18A, BID, CHEK2, and CEACAM4) was identified employing multivariate cox analysis. Moreover, the risk model was created utilizing the subsequent formula: risk factor = KIF18A ×0.5799 + BID × 0.6975 + CHEK2 × 0.5761 + CEACAM4 × 0.5914. The subjects were categorized into two groups, namely high- and low-risk, based on the median value of the risk score.

### 3.3 Validation of the ARGs prognostic signature

**Figure 4:**
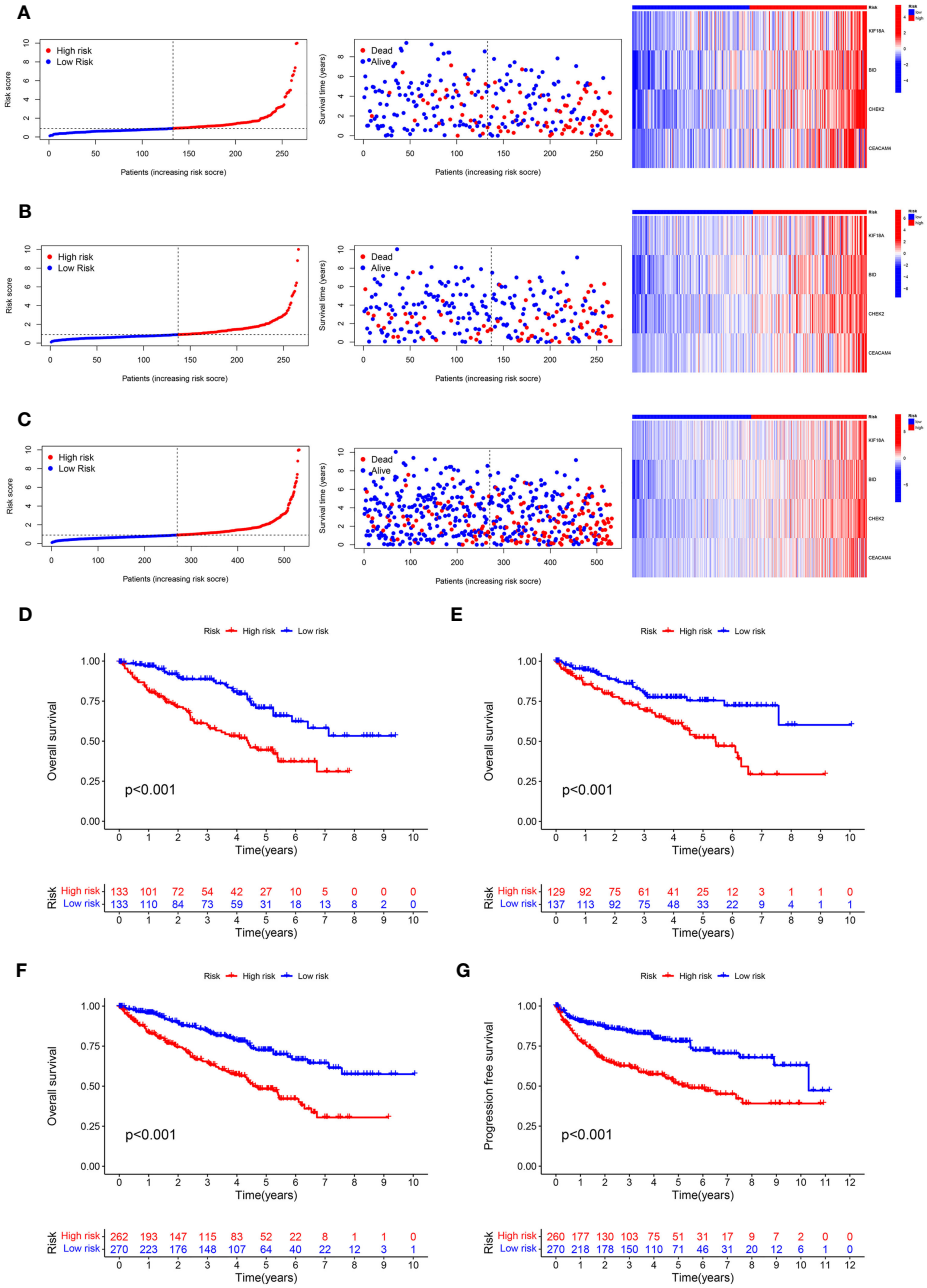
Predictive value of the ARS. **(A–C)** Distribution plots of the risk score, overall survival (OS) status, and heatmap of gene expressions of the 4 prognostic ARGs in training set, test set and entire set. **(D–F)** Kaplan–Meier curves of survival outcome between high- and low-risk groups in the training set, test set and entire set. **(G)** Kaplan–Meier survival curve showed that the progression free survival of the high-risk group was significantly shorter than that of the low-risk group in the entire set.

As displayed in **Figures 4A–C**, the scatter dot plot distinguished the risk score and clinical status of KIRC samples. Based on the heatmap, the KIF18A, CHEK2, BID, and CEACAM4 expressions were considerably greater in the high-risk group in both cohorts. The Kaplan-Meier survival curves indicated that in both cohorts, patients in the high-risk group had poor clinical outcomes (**Figures 4D–F**). Moreover, in the entire cohort, Kaplan-Meier survival analysis revealed that patients in the low-risk group presented considerably better progression-free survival (**Figure 4G**). The outcomes demonstrated that the prognostic signature based on ARGs can offer a dependable evaluation of the prognosis for KIRC.

### 3.4 The risk model based on ARGs prognostic signature was an independent prognosis indicator

**Figure 5:**
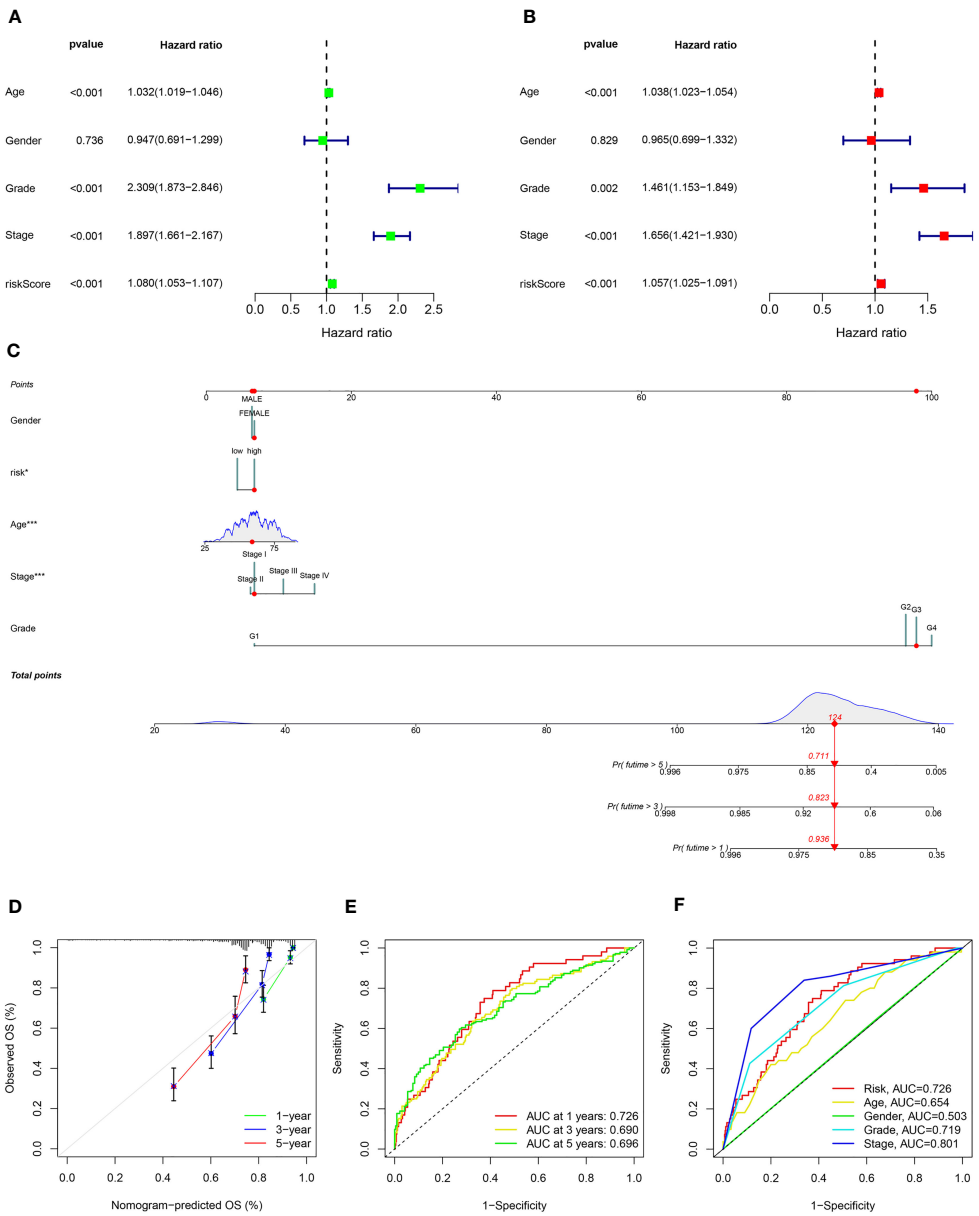
Independent prognosis analysis of the ARS. **(A)** Univariate Cox regression analysis and **(B)** multivariate Cox regression analysis to estimate the independence of the ARS. **(C)** Nomogram construction based on the ARS and clinicopathological characteristics. **(D)** Calibration curves shows the concordance between predicted and observed 1, 3 and 5-years survival rates. **(E)** Time-dependent ROC curve shows the AUC at 1-, 3-, and 5-year. **(F)** ROC curve shows the accuracy of the risk score and clinicopathological characteristics.

The univariate and multivariate cox regression analyses were used to assess the independence of the risk model. Univariate Cox regression analysis revealed that age (hazard ratio (HR) = 1.032, *P*< 0.001), grade (HR = 2.309, *P*< 0.001), stage (HR = 1.897, *P*< 0.001), and risk score (HR = 1.080, *P*< 0.001) were all highly associated with overall survival (OS) rate in KIRC (**Figure 5A**). After conducting a multivariate Cox analysis, it was discovered that the risk score remained statistically significant, indicating that the prognostic signature proposed using ARGs could be independent of other clinical factors (as depicted in **Figure 5B**). To capitalize on the signature’s prognosis value, a novel nomogram was developed by integrating clinical factors and a risk model for precisely predicting the 1-, 3-, and 5-year survival possibility of KIRC (**Figure 5C**). The calibration plots for predicting survival rates at 1, 3, and 5 years demonstrated that the nomogram possessed considerable accuracy in predicting the prognosis (as indicated in **Figure 5D**). Moreover, the prognostic model based on ARGs demonstrated strong predictive ability for 1-, 3-, and 5-year overall survival (OS) based on the high AUC values in the time-dependent ROC analysis (**Figure 5E**). The ROC curve analysis implied that the ARGs prognostic signature had an AUC of 0.726, indicating a stable and reliable predictive ability (**Figure 5F**). The findings suggested that a risk score calculated using ARGs was found to be an independent predictor of prognosis, accurately evaluating the survival probability of KIRC patients relative to clinicopathological characteristics.

### 3.5 Role of the signature for OS in the prognosis of KIRC stratified by clinicopathological variables

**Figure 6:**
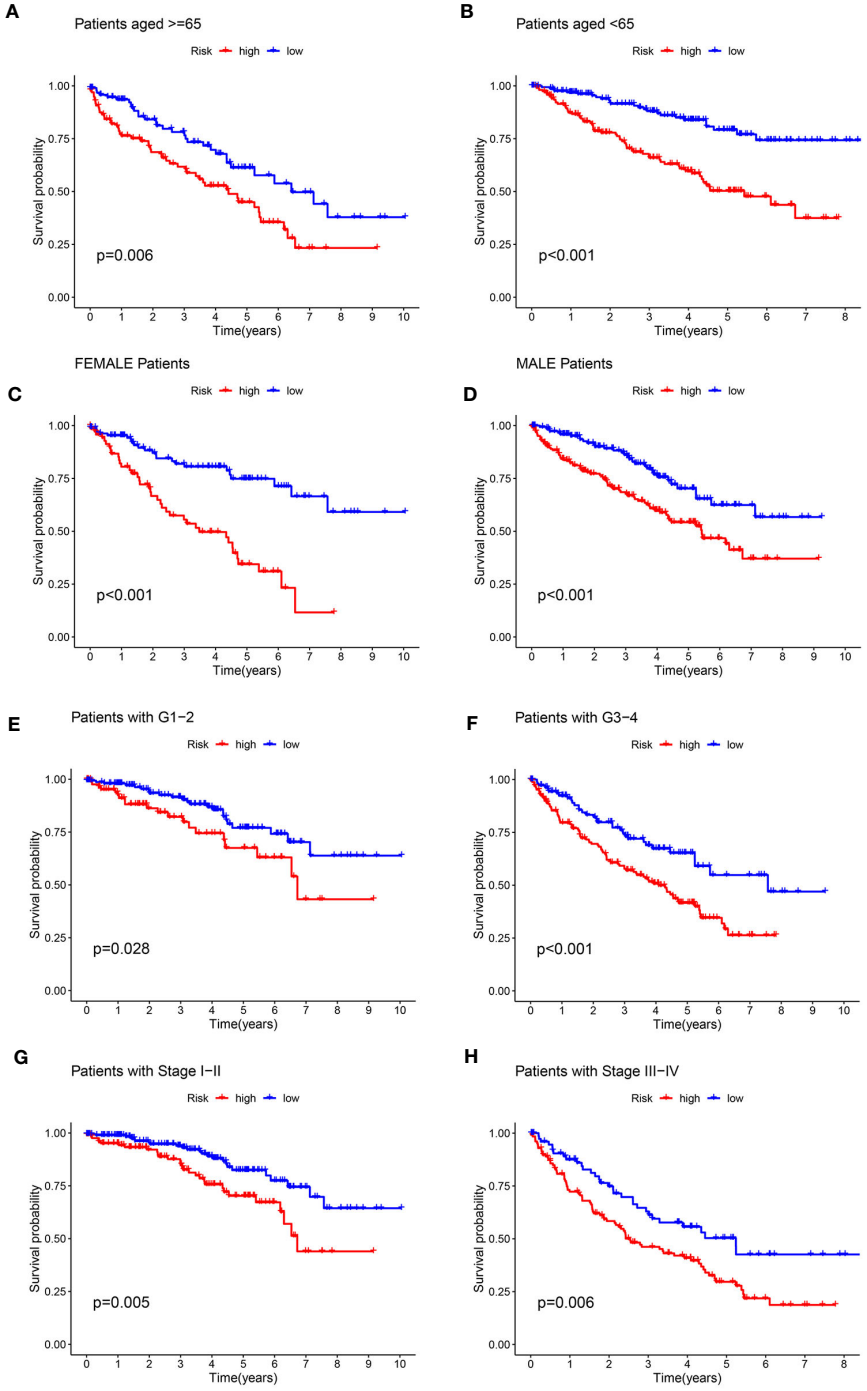
Kaplan-Meier survival curves for the high- and low-risk groups stratified by clinicopathological variables. **(A, B)** Age; **(C, D)** Gender; **(E, F)** Grade; **(G, H)** Stage.

To evaluate the prognostic significance of the ARGs signature in predicting overall survival (OS) in KIRC patients based on clinicopathological characteristics, the ccRCC patients were stratified into high- and low-risk groups among various clinical and pathological features. Across all the stratifications, the low-risk group consistently exhibited considerably higher rates of OS in comparison to the high-risk group, as illustrated in **Figure 6**. The study demonstrated that the anoikis-related signature for OS can provide an accurate prediction for the prognosis of KIRC, irrespective of the clinicopathological characteristic.

### 3.6 Pathway and process enrichment analyses

**Figure 7:**
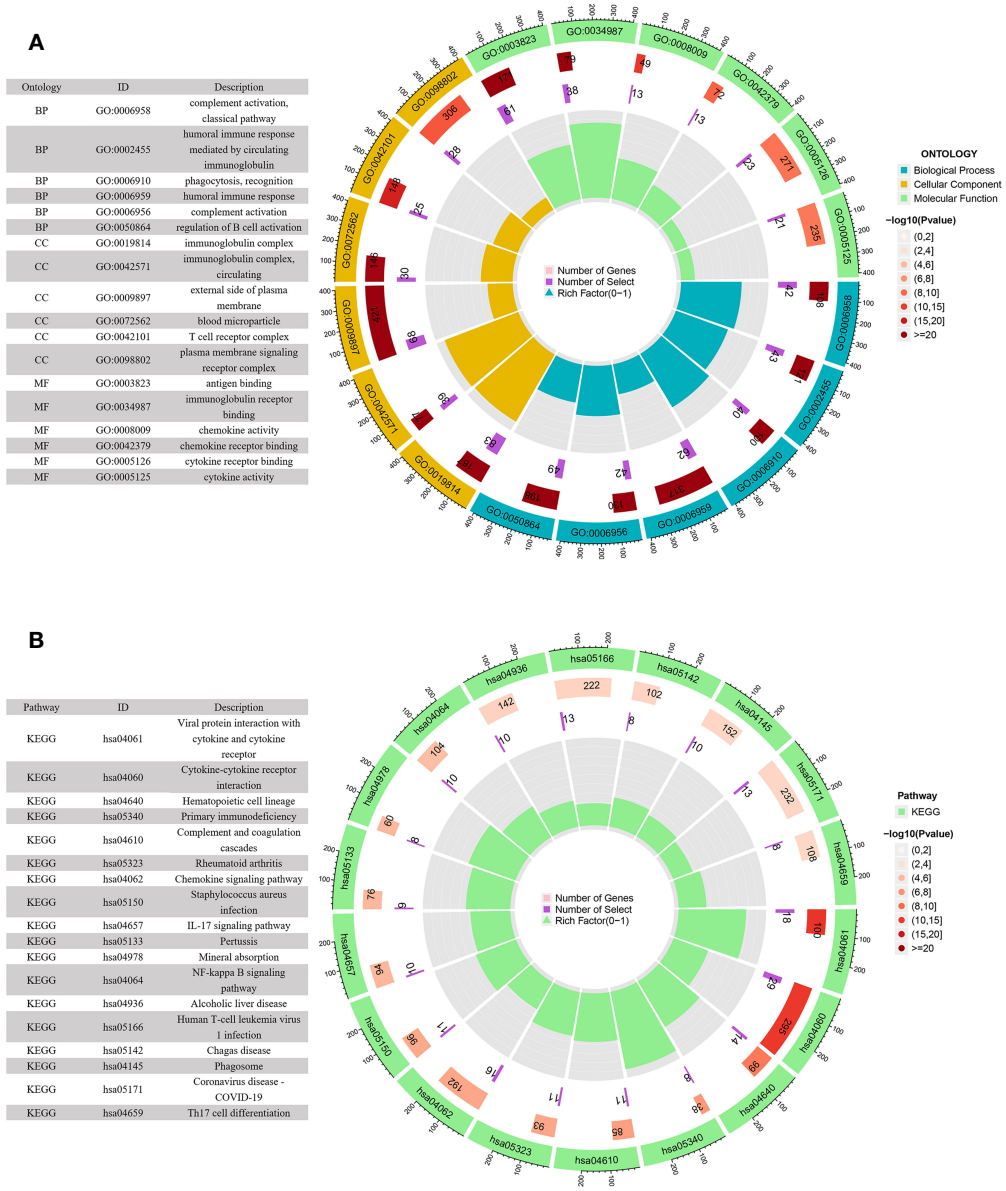
GO and KEGG enrichment analyses of DEGs in the low- and high-risk groups. **(A)** GO enrichment circle diagram of DEGs. **(B)** KEGG enrichment circle diagram of DEGs.

KEGG pathway and GO enrichment analyses were conducted to investigate the biological functions and pathways of genes that were differentially expressed between the high-risk and low-risk groups. GO analysis demonstrated enrichment of immune-related molecular processes such as humoral immune response, complement activation, and regulation of B cell activation (**Figure 7A**). Meanwhile, KEGG pathway studies revealed that DEGs were abundant in the primary immunodeficiency, complement and coagulation cascades, cytokine-cytokine receptor interaction, IL-17 signaling pathway, and chemokine signaling pathway (**Figure 7B**). These findings showed that the function of the ARGs in the tumorigenesis of ccRCC may be regulated by immune-related signaling pathways.

### 3.7 Immune status difference between patients with high-risk and low-risk KIRC

**Figure 8:**
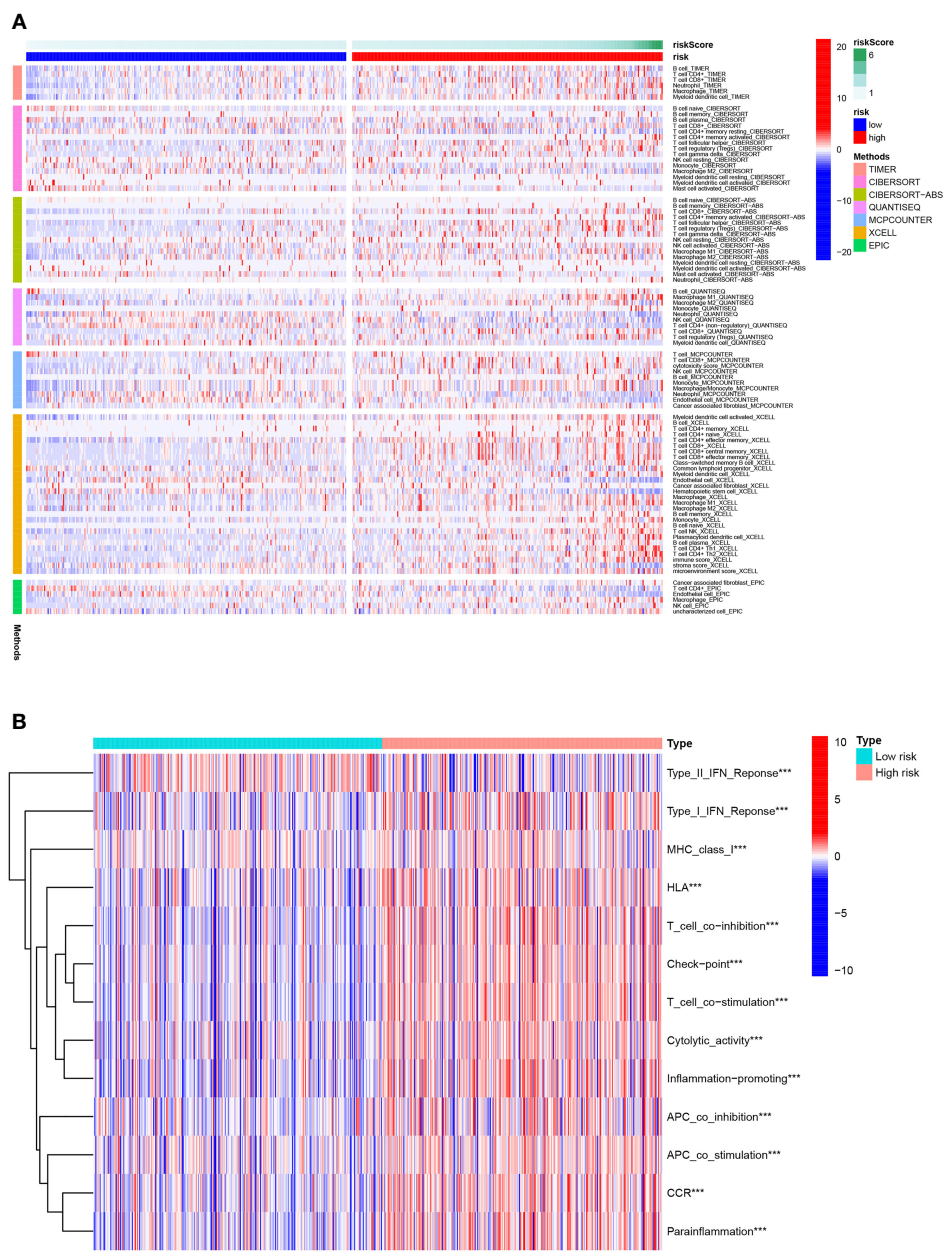
Immune status difference between high- and low-risk groups. **(A)** Heatmap for the tumor-infiltrating immune cells by different algorithms among high- and low-risk groups. **(B)** Immune-related function between high- and low-risk groups. ***P< 0.001.

**Figure 9:**
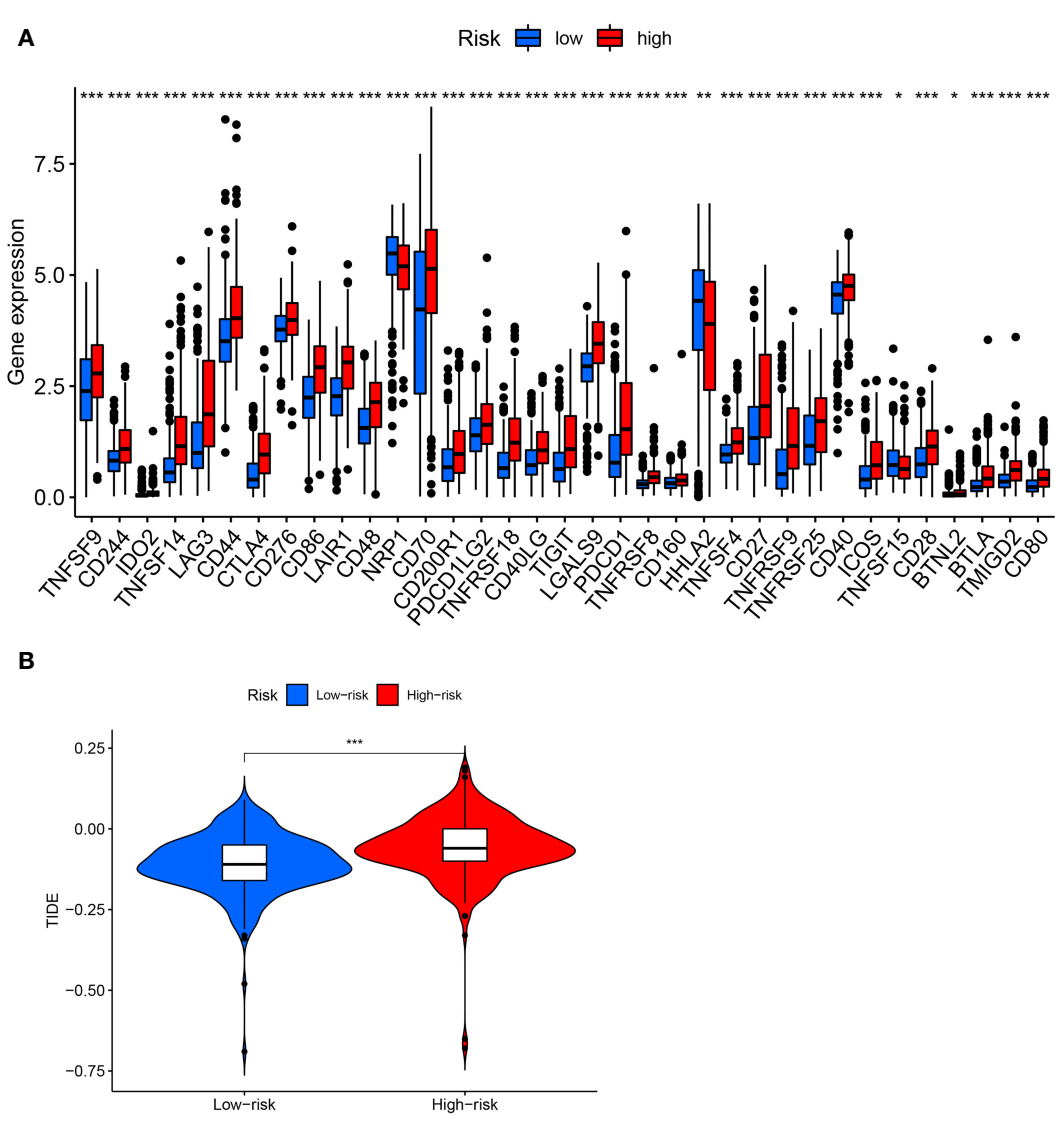
Analysis of immune checkpoints and immunotherapeutic response. **(A)** Expression of immune checkpoints between high- and low-risk groups. **(B)** TIDE score in two risk groups. * P< 0.05; ** P< 0.01; *** P< 0.001.

**Figure 8A** depicted an immune response heatmap based on several enrichment analysis algorithms. The relationship of immune cell subsets and associated functions was examined using ssGSEA, and the results revealed considerable differences in IFN response, MHC, HLA, T cell functions, checkpoint, cytolytic activities, APC, CCR, promoting inflammation, and para-inflammation between the low-risk group and high-risk group (**Figure 8B**). Additionally, our findings revealed a substantial difference in the gene expression of immune checkpoints such as TNFRSF9, CD244, IDO2, and PDCD1 between the two groups (**Figure 9A**). Because the immune infiltration landscape for individuals with ccRCC differs noticeably, the efficacy of immunotherapy in patients with high-risk and low-risk conditions was further examined. TIDE results revealed that patients with a high-risk score had a greater TIDE score, indicating that the low-risk group reacted better to immunotherapy in comparison to high-risk group (**Figure 9B**).

### 3.8 The relationship between risk signature and somatic mutation

**Figure 10:**
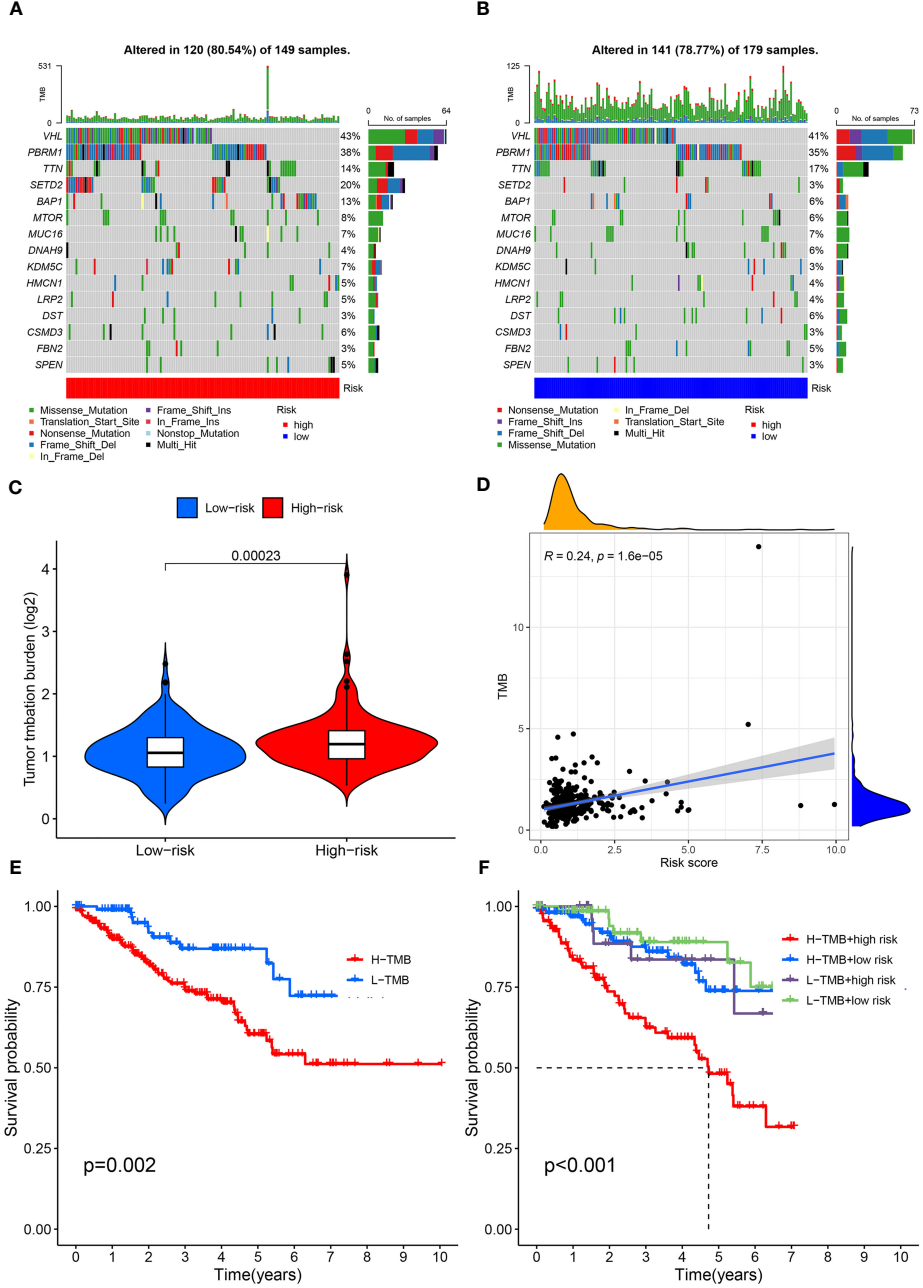
Mutation profile and relationship between tumor mutation burden (TMB) and risk score. **(A)** Mutation profile of the high-risk group. **(B)** Mutation profile of the low-risk group. **(C)** TMB differences in the high- and low-risk groups. **(D)** Positive correlation between tumor mutation burden and the risk score. **(E)** Association of overall survival and TMB in ccRCC patients. **(F)** Association of overall survival and TMB combined with risk score in ccRCC patients.

First, the somatic variation driver genes of the low-risk and high-risk groups were compared. The top 15 driver genes with the highest mutation frequency were shown using a waterfall diagram. Accordingly, it was discovered that the prevalence of these gene mutations was generally greater in the high-risk group compared to the low-risk group (**Figures 10A, B**). Subsequently, the tumor mutation burden (TMB) was examined and the TMB of the high-risk group was substantially greater than the low-risk group, as shown by the result (*P*< 0.001) (**Figure 10C**). We found a highly significant positive relationship between the risk score and TMB (Spearman coefficient: r = 0.24, *P*< 0.001) (**Figure 10D**). Furthermore, we examined the TMB impact on prognosis. Based on the results, individuals in the high-TMB group exhibited a less favorable prognosis compared to those in the low-TMB group (*P* = 0.002) (**Figure 10E**). TMB and risk score were assessed for their impact on prognostic stratification due to their synergistic effect. As revealed by the outcomes, in both low- and high-TMB groups, the survival difference of risk score subtypes were significant. (*P*< 0.001) (**Figure 10F**). The findings may present new strategies for exploring targeted therapy and immunotherapy from the perspectives of gene mutation and anoikis.

### 3.9 Investigation of the four ARGs expression in KIRC

**Figure 11:**
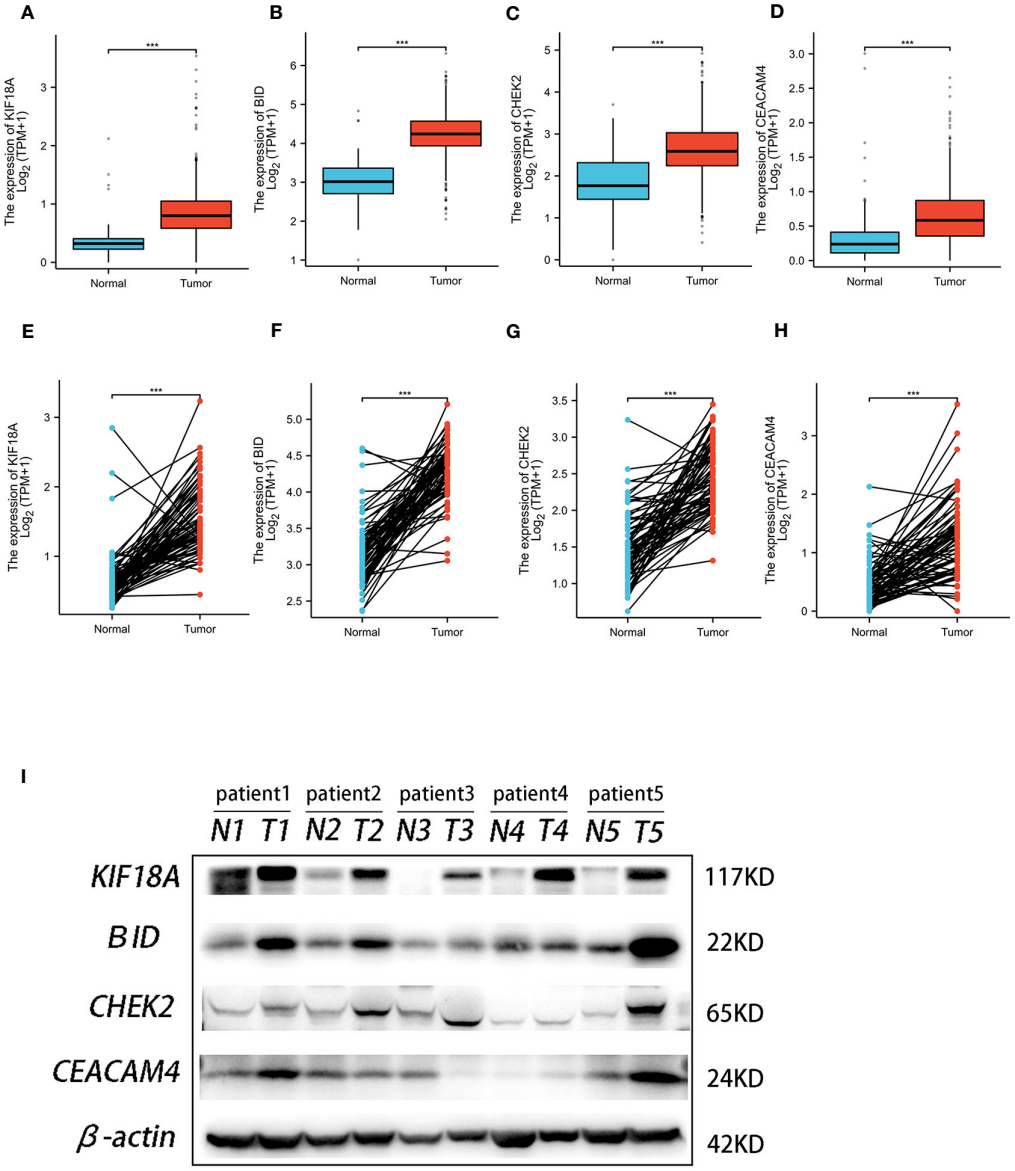
The expression of the four genes in this signature. Expression of KIF18A **(A)**, BID **(B)**, CHEK2 **(C)**, and CEACAM4 **(D)** in ccRCC and normal samples from TCGA and GTEx. Expression of KIF18A **(E)**, BID **(F)**, CHEK2 **(G)**, and CEACAM4 **(H)** in paired ccRCC and adjacent normal tissue samples from TCGA. **(I)** Western blot analysis was conducted to examine the protein expression of four genes among ccRCC tissues and matched adjacent normal tissues. *** P< 0.001.

Analysis of TCGA and GTEx data revealed significantly lower expression of the four ARGs in normal kidney tissues compared to ccRCC samples (**Figures 11A–D**). Furthermore, paired analysis showed higher expression of these four genes in renal cancer tissues versus matched normal adjacent tissues (**Figures 11E–H**). Western blot validation in five ccRCC and paired paracancer tissues confirmed the mRNA results, with increased protein levels of the four ARGs in ccRCC compared to paracancer tissues (**Figure 11I**).

## 4 Discussion

ccRCC is defined as the most prevalent histological variant of RCC, which is likely to have an unsatisfactory outcome due to a lack of trustworthy biomarkers connected to prognosis (12). To increase the survival duration of ccRCC patients, early diagnosis, and risk stratification are crucial. Anoikis is a form of programmed cell death that is initiated to eliminate detached cells through activation of both extrinsic and intrinsic apoptotic pathways (13). Cancer cells use a variety of defense mechanisms to combat anoikis, which enhances their invasiveness and metastasis potential (14–16). Recent clinical retrospective research also demonstrated that anoikis resistance was linked to lymph node metastasis and a poor prognosis (17). Hence, ARGs have the potential to be therapeutic targets as well as tumor prognostic indicators.

Herein, we first employed a combined analysis of the TCGA-KIRC and GeneCards datasets to screen out differentially expressed ARGs in ccRCC. After conducting stepwise cox regression, we constructed a novel predictive model consisting of four ARGs (KIF18A, BID, CHEK2, and CEACAM4). Each patient in both groups had a signature-correlated risk score, which was divided by the KIRC cohort’s median risk score to establish whether they were in the high-risk or low-risk category. The prognosis between the two groups differs noticeably. Besides, we generated a nomogram that improved the accuracy of the risk model by combining age, gender, grade, stage, and risk score. The calibration diagrams indicated that the nomogram was well-suited for predicting the prognosis.

Above, 4 ARGs of our risk model were identified as connected to the ccRCC OS rate. These genes have all been shown to be closely related to tumors. KIF18A belongs to the kinesin superfamily. The primary role of KIF18A is to influence dynamics at the plus end of kinetochore microtubules, ensuring accurate chromosomal placement and spindle tension (18). The transcriptional and translational levels of KIF18A were both increased in lung adenocarcinoma. In lung adenocarcinoma cells, KIF18A knockdown caused apoptosis and G2/M phase arrest, as well as a reduction in their capability for proliferating both *in vitro* and *in vivo* (19). In hepatoma cells, silencing KIF18A reduced the expression of cyclin B1, MMP-9, MMP-7, and Akt-related proteins and inhibited hepatoma cell growth, invasion, and metastasis (20). A higher level of KIF18A expression in ccRCC promoted cell proliferation and was indicative of a poor prognosis (21). These data suggested that KIF18A facilitated the development of malignant traits in certain tumors, which was consistent with our findings that KIF18A was abundantly expressed in high-risk cohorts. BID is a member of the Bcl-2 protein family, responsible for controlling the permeability of the outer membrane of mitochondria, which is a critical step in the process of apoptosis (22). According to a recent study, truncated BID controls the cisplatin response in ovarian cancer by triggering the mitochondrial apoptosis pathway (23). Genome stability is essential to preventing cancer risk caused by genes with inherited loss-of-function variants in DNA damage recovery and genome integrity checkpoint control (24). A well-known illustration is the checkpoint kinase 2 (CHEK2) gene, which produces the serine-threonine kinase protein CHK2 that controls cell-cycle progression and apoptosis when DNA damage occurs (25). The analysis of function revealed that harmful missense variants in CHEK2 were linked to a higher risk of cancer (26). Another study found that germline CHEK2 mutations have a significant correlation with ovarian and breast cancer (27). An investigation to observe how common germline mutations are in genes that contribute to cancer susceptibility among patients with advanced renal cell carcinoma revealed that out of 254 patients, 41 (16.1%) had germline mutations, and the most frequent mutations were found in the CHEK2 gene (28). These results demonstrated that certain anoikis-related gene mutations were connected to an increased risk of RCC. CEACAM4 is a member of the CEACAM subfamily of the immunoglobulin superfamily. For decades, CEACAM5 (CEA), a member of the same superfamily, has been used as a biomarker to observe the progression of several types of cancer following surgery (29). At the moment, significant progress has been made with CEA-targeted cancer immunotherapies such as bispecific antibodies for radioimmunotherapy and imaging, chimeric antigen receptor T cells, and bispecific T cell engagers (30). These findings have significant ramifications for comprehending the complex biology of CEACAMs in both normal and malignant tissues as well as their novel function in immunotherapy for tumor.

It is believed that RCC is an immunogenic tumor. In recent times, immunotherapy has emerged as a novel treatment option for this type of cancer (31). TMB is emerging as a potential biomarker among various cancer types to predict immune checkpoint inhibitor efficacy (32). Many studies have demonstrated a significant association between immunotherapy response and TMB (33–35). Therefore, we further examined the efficacy of this signature as applied to the immune and TMB factors of ccRCC. Firstly, we compared immune cell infiltration and immune function in both high- and low-risk groups. In comparison to the low-risk group, the high-risk group exhibited a higher level of infiltration by immune cells, including B cells, CD8+T cells, CD4+T cells, macrophages, neutrophils, and myeloid dendritic cells. Additionally, immune functions in the high-risk group were stronger in comparison to that in the low-risk group, demonstrating that the high-risk group displayed increased anti-tumor immune activity compared to the low-risk group. Further analysis was conducted to see if this signature could be used as a reference for immunotherapy response. We examined the expression levels of immune checkpoints, which are the classical molecules utilized for assessing the effectiveness of immunotherapy. The result showed that most immune checkpoints had considerably greater expression levels in the high-risk group in comparison to the low-risk group. Moreover, we discovered that the TIDE score is lower in the low-risk group, implying that immunotherapy could be ineffective for patients in the high-risk group. Lastly, we investigated the connection between the risk score of the prognosis signature and TMB. There was a positive correlation between the risk score and TMB, and the high-risk group patients showed a tendency to have higher TMB than patients in the low-risk group. Meanwhile, we analyzed the TMB impact on prognosis. As indicated by the results, high TMB might be related to poorer outcomes, which is line with previous studies (36–39).

Although the efficacy of our suggested signature in predicting the prognosis of KIRC patients is impressive, there are several limitations in the current project. To begin with, the TCGA dataset was the only source used to gather clinical cohorts of KIRC cases, requiring the use of external data to verify the results. In addition, further biochemical studies with ARGs in KIRC, both *in vitro* and *in vivo*, are necessary.

To summarize, we developed a robust gene signature in KIRC. This anoikis-related signature can be employed as an independent prognostic factor in ccRCC patients to identify immune conditions and evaluate immunotherapy response. Our research offers new idea and understandings of a new prospective clinical approach for KIRC patients.

## Data availability statement

Publicly available datasets were analyzed in this study. The original contributions presented in the study are included in the Jianguoyun Raw Data link (https://www.jianguoyun.com/p/DWzF5wcQlIXMCxies54FIAA), further inquiries can be directed to the corresponding author.

## Ethics statement

The study was approved by the ethical committee of Ningbo Yinzhou No.2 Hospital (2023-KY-003) and followed the guidelines of the Declaration of Helsinki. Written informed consent was obtained from all individual patients included in the study. The studies were conducted in accordance with the local legislation and institutional requirements. The participants provided their written informed consent to participate in this study.

## Author contributions

Conceptualization: QW, ML. Methodology: QW, XQ, SH. Formal analysis and investigation: QW, YS. Writing - original draft preparation: QW. Writing - review and editing: ML, XW. Funding acquisition: GW. Resources: SH, XQ. Supervision: GW. All authors commented on previous versions of the manuscript. All authors contributed to the article and approved the submitted version.
