# Supplementary material for: Development and validation of a novel anoikis-related gene signature in clear cell renal cell carcinoma
Source: Front Oncol. 2023 Oct 26;13:1211103. doi: 10.3389/fonc.2023.1211103 (PMC10641395; doi:10.3389/fonc.2023.1211103)
Supplement: Supplementary file 1 [file Table_1.docx]

Supplementary Table S1 The list of 496 anoikis-related genes (ARGs).

| Gene Symbol | Relevance score |
| --- | --- |
| BRMS1 | 14.70989609 |
| PTK2 | 7.294509888 |
| NTRK2 | 7.267454624 |
| BCL2L11 | 6.69656086 |
| SRC | 6.190118313 |
| CEACAM6 | 6.106050968 |
| CAV1 | 5.468391895 |
| AKT1 | 5.412473679 |
| ITGB1 | 5.007508755 |
| CEACAM5 | 4.65794754 |
| EGFR | 4.620491028 |
| BCL2 | 4.545550346 |
| CASP8 | 4.493535042 |
| SIK1 | 4.376661777 |
| PTRH2 | 4.196393013 |
| STAT3 | 4.145073891 |
| TLE1 | 4.072757244 |
| DAPK2 | 3.989923239 |
| CTNNB1 | 3.979063034 |
| ZNF304 | 3.941507101 |
| MAPK1 | 3.738428116 |
| BMF | 3.735781193 |
| ITGA5 | 3.680768967 |
| TP53 | 3.643580914 |
| MCL1 | 3.582160473 |
| BCL2L1 | 3.364901543 |
| CASP3 | 3.123229027 |
| CDH1 | 3.062824488 |
| BAD | 2.966172934 |
| PIK3CA | 2.944177628 |
| PAK1 | 2.933961391 |
| ITGAV | 2.878329039 |
| FN1 | 2.824431419 |
| MAPK3 | 2.732716084 |
| PTGS2 | 2.694960117 |
| BAX | 2.550824881 |
| BCAR1 | 2.550824881 |
| PTEN | 2.521120548 |
| ERBB2 | 2.436502695 |
| PDK4 | 2.415143013 |
| ANGPTL4 | 2.400918961 |
| CYCS | 2.34193635 |
| BRAF | 2.337760448 |
| YAP1 | 2.334618092 |
| ANKRD13C | 2.329473257 |
| ITGA2 | 2.300601006 |
| ANXA5 | 2.268351793 |
| BIRC5 | 2.258077145 |
| MTOR | 2.25141716 |
| TIMP1 | 2.247738838 |
| BDNF | 2.223801374 |
| CSPG4 | 2.197484732 |
| BSG | 2.197484732 |
| AKT2 | 2.18487072 |
| STK11 | 2.151944637 |
| IGF1 | 2.150004625 |
| IGF1R | 2.149197817 |
| ITGA6 | 2.112035513 |
| ILK | 2.085387707 |
| CFLAR | 2.085190296 |
| RHOA | 2.070759058 |
| HIF1A | 2.067117691 |
| DAP3 | 2.057498455 |
| MYBBP1A | 2.02230978 |
| ITGA3 | 2.000199795 |
| TLE5 | 1.999629498 |
| PTK2B | 1.99717474 |
| CCND1 | 1.983445644 |
| CTTN | 1.983445644 |
| CALR | 1.946776748 |
| ATF4 | 1.946776748 |
| CDCP1 | 1.933939457 |
| SKP2 | 1.909861207 |
| CHEK2 | 1.908558011 |
| HGF | 1.885339022 |
| E2F1 | 1.881289601 |
| EGF | 1.872653484 |
| PIK3CG | 1.8694067 |
| ITGB4 | 1.857610226 |
| DAPK1 | 1.847485065 |
| PIK3R1 | 1.822576404 |
| PIK3R3 | 1.816892266 |
| MAP2K1 | 1.798025489 |
| CXCL12 | 1.778746605 |
| LGALS3 | 1.743283868 |
| FBXW7-AS1 | 1.735649347 |
| BAK1 | 1.731907725 |
| ABHD4 | 1.708241105 |
| CD44 | 1.704720378 |
| ITGA4 | 1.691461802 |
| FADD | 1.691461802 |
| PHLDA2 | 1.691461802 |
| TGFB1 | 1.687233329 |
| HMCN1 | 1.687233329 |
| MMP2 | 1.674071789 |
| CEBPB | 1.674071789 |
| CEMIP | 1.674071789 |
| CDKN3 | 1.668370247 |
| CBL | 1.655999064 |
| CASP9 | 1.655999064 |
| SFN | 1.655999064 |
| MTDH | 1.655999064 |
| PRKCA | 1.637156248 |
| TNFRSF10B | 1.637156248 |
| CXCL8 | 1.637156248 |
| MIR200C | 1.637156248 |
| AR | 1.620118499 |
| CDKN2A | 1.617435694 |
| MAPK8 | 1.617435694 |
| CPT1A | 1.617435694 |
| PIK3CB | 1.617435694 |
| CLDN1 | 1.617435694 |
| MIR204 | 1.617435694 |
| MIR26A1 | 1.617435694 |
| CDKN1A | 1.596701741 |
| CDKN1B | 1.596701741 |
| KLF12 | 1.596701741 |
| NTRK1 | 1.575224042 |
| PLAU | 1.574780226 |
| MYC | 1.574780226 |
| PLK1 | 1.574780226 |
| SMAD4 | 1.574780226 |
| MUC1 | 1.574780226 |
| PLAUR | 1.574780226 |
| LGALS1 | 1.574780226 |
| PYCARD | 1.574780226 |
| SESN2 | 1.574780226 |
| ITGB3 | 1.570110321 |
| KRAS | 1.570110321 |
| THBS1 | 1.55144012 |
| BID | 1.55144012 |
| HRAS | 1.536660552 |
| CDK11B | 1.526364088 |
| CDK11A | 1.526364088 |
| XIAP | 1.519712329 |
| PPARG | 1.499095678 |
| IL6 | 1.499095678 |
| MIR145 | 1.499095678 |
| CCR7 | 1.468933702 |
| MSLN | 1.468933702 |
| RAC1 | 1.463076115 |
| GRHL2 | 1.463076115 |
| NOTCH1 | 1.438928962 |
| RHOG | 1.437688351 |
| CCAR2 | 1.437688351 |
| NQO1 | 1.434698343 |
| BIRC3 | 1.432427526 |
| MMP13 | 1.400075436 |
| FAS | 1.39708066 |
| MTA1 | 1.39708066 |
| MYO5A | 1.394088984 |
| EDA2R | 1.394088984 |
| CCN6 | 1.394088984 |
| MMP9 | 1.375791311 |
| ABL1 | 1.375791311 |
| MAPK11 | 1.375791311 |
| PTHLH | 1.372004628 |
| PDGFB | 1.355057478 |
| GLI2 | 1.355057478 |
| EZH2 | 1.353923321 |
| CXCR4 | 1.344736099 |
| RIPK1 | 1.336065769 |
| HMGA1 | 1.333135962 |
| SIK2 | 1.333135962 |
| TNFSF10 | 1.333135962 |
| ANGPTL2 | 1.314574242 |
| S100A4 | 1.309795856 |
| ETV4 | 1.309795856 |
| NTF3 | 1.309795856 |
| MIR21 | 1.309795856 |
| MIR124-1 | 1.309795856 |
| HTRA1 | 1.284719825 |
| LATS1 | 1.284719825 |
| CEACAM3 | 1.284719825 |
| EIF2AK3 | 1.281760216 |
| LAMC2 | 1.281760216 |
| LAMA3 | 1.281760216 |
| LAMB3 | 1.281760216 |
| CDH2 | 1.262039661 |
| CSNK2A1 | 1.262039661 |
| EDIL3 | 1.262039661 |
| ZEB2 | 1.257451415 |
| TLN1 | 1.257451415 |
| EPHA2 | 1.241305828 |
| SOD2 | 1.241305828 |
| SIRT3 | 1.241305828 |
| OLFM3 | 1.241305828 |
| CLU | 1.227289438 |
| SPINK1 | 1.227289438 |
| CPEB2 | 1.227289438 |
| NAT1 | 1.219384193 |
| TSG101 | 1.219384193 |
| MIR200A | 1.219384193 |
| MIR6744 | 1.219384193 |
| SERPINA1 | 1.213397741 |
| AKT3 | 1.196044087 |
| RELA | 1.196044087 |
| TNFRSF1A | 1.196044087 |
| AFP | 1.196044087 |
| FASLG | 1.196044087 |
| EEF1A1 | 1.196044087 |
| ITGA8 | 1.196044087 |
| SATB1 | 1.196044087 |
| CD63 | 1.196044087 |
| LTB4R2 | 1.196044087 |
| NOX4 | 1.196044087 |
| PBK | 1.196044087 |
| MAVS | 1.196044087 |
| HRC | 1.196044087 |
| RHOB | 1.19305408 |
| CCN2 | 1.19305408 |
| PPP1R13B | 1.19305408 |
| PLG | 1.186806202 |
| MET | 1.185467005 |
| RAF1 | 1.170968175 |
| PARP1 | 1.170968175 |
| PRKCQ | 1.170968175 |
| BRCA2 | 1.170968175 |
| RB1 | 1.170968175 |
| DOCK1 | 1.170968175 |
| HAVCR2 | 1.170968175 |
| SP1 | 1.170968175 |
| VTN | 1.170968175 |
| INHBB | 1.170968175 |
| PDCD4 | 1.170968175 |
| PRPF4B | 1.170968175 |
| RANBP9 | 1.170968175 |
| SESN1 | 1.170968175 |
| SESN3 | 1.170968175 |
| ZBTB7A | 1.170968175 |
| CD24 | 1.170968175 |
| MIR141 | 1.170968175 |
| ELANE | 1.15244472 |
| KDR | 1.143699646 |
| MDM2 | 1.143699646 |
| NFE2L2 | 1.143699646 |
| PRKCI | 1.143699646 |
| ZEB1 | 1.143699646 |
| HK2 | 1.143699646 |
| KL | 1.143699646 |
| CRYAB | 1.143699646 |
| EPHB6 | 1.143699646 |
| FGF2 | 1.143699646 |
| LTF | 1.143699646 |
| IQGAP1 | 1.143699646 |
| MGAT5 | 1.143699646 |
| SDCBP | 1.143699646 |
| ABHD2 | 1.143699646 |
| SPIB | 1.143699646 |
| TRIM31 | 1.143699646 |
| MIR1827 | 1.143699646 |
| PDGFRB | 1.113537788 |
| TLR3 | 1.113537788 |
| PLAT | 1.113537788 |
| ROCK1 | 1.113537788 |
| NRAS | 1.113537788 |
| CASP10 | 1.113537788 |
| PAK4 | 1.113537788 |
| VEGFA | 1.113537788 |
| PIN1 | 1.113537788 |
| YWHAZ | 1.113537788 |
| TWIST1 | 1.113537788 |
| UBE2C | 1.113537788 |
| IL1RAP | 1.113537788 |
| BMP6 | 1.113537788 |
| ELK1 | 1.113537788 |
| PRDX4 | 1.113537788 |
| BNIP3 | 1.113537788 |
| BNIP3L | 1.113537788 |
| KDM3A | 1.113537788 |
| LMO3 | 1.113537788 |
| ZNF32 | 1.113537788 |
| MIR200B | 1.113537788 |
| MIR525 | 1.113537788 |
| MIR363 | 1.113537788 |
| TUBB3 | 1.099521399 |
| HSP90B1 | 1.099521399 |
| PTPN11 | 1.07930243 |
| SLC2A1 | 1.07930243 |
| HMOX1 | 1.07930243 |
| PRKACA | 1.07930243 |
| PAK3 | 1.07930243 |
| PIK3R2 | 1.07930243 |
| PPP2CA | 1.07930243 |
| CASP6 | 1.07930243 |
| CD36 | 1.07930243 |
| CDH3 | 1.07930243 |
| LRP1 | 1.07930243 |
| PTK6 | 1.07930243 |
| EEF2K | 1.07930243 |
| GLO1 | 1.07930243 |
| LPAR1 | 1.07930243 |
| PAK2 | 1.07930243 |
| ADCY10 | 1.07930243 |
| RBL2 | 1.07930243 |
| CEACAM1 | 1.07930243 |
| GDF2 | 1.07930243 |
| SIRPA | 1.07930243 |
| TRAF2 | 1.07930243 |
| APOBEC3G | 1.07930243 |
| MNX1 | 1.07930243 |
| TNFRSF12A | 1.07930243 |
| VPS37A | 1.07930243 |
| BAG1 | 1.07930243 |
| IL17A | 1.07930243 |
| COL13A1 | 1.07930243 |
| RAD9A | 1.07930243 |
| IFI27 | 1.07930243 |
| MEGF11 | 1.07930243 |
| ITPRIP | 1.07930243 |
| BCL2L15 | 1.07930243 |
| SNAI2 | 1.050556302 |
| GLUD1 | 1.038692951 |
| NOTCH3 | 1.038692951 |
| PTPN1 | 1.038692951 |
| FASN | 1.038692951 |
| MYH9 | 1.038692951 |
| RPS6KB1 | 1.038692951 |
| SIRT1 | 1.038692951 |
| TPM1 | 1.038692951 |
| PPP2R1A | 1.038692951 |
| COL4A2 | 1.038692951 |
| CTNND1 | 1.038692951 |
| MMP11 | 1.038692951 |
| CD151 | 1.038692951 |
| PPP2R2A | 1.038692951 |
| SEMA7A | 1.038692951 |
| ARHGEF7 | 1.038692951 |
| BST2 | 1.038692951 |
| PPP2R5A | 1.038692951 |
| PPP2R2D | 1.038692951 |
| CCN1 | 1.038692951 |
| CCDC178 | 1.038692951 |
| MIR10A | 1.038692951 |
| MIR30C1 | 1.038692951 |
| MIR30B | 1.038692951 |
| SHC1 | 1.01773119 |
| BUB1 | 0.985769749 |
| CDC25C | 0.985769749 |
| BUB3 | 0.985769749 |
| FER | 0.985769749 |
| ITGB5 | 0.985769749 |
| SETD2 | 0.985769749 |
| TP73 | 0.985769749 |
| CDK1 | 0.985769749 |
| SLCO1B3 | 0.985769749 |
| BCL2L2 | 0.985769749 |
| MAD2L1 | 0.985769749 |
| DLG1 | 0.985769749 |
| PDCD6IP | 0.985769749 |
| EDAR | 0.985769749 |
| SCRIB | 0.985769749 |
| SH3GLB1 | 0.985769749 |
| TDGF1 | 0.985769749 |
| DYNLL2 | 0.985769749 |
| TSC2 | 0.958890557 |
| BAG4 | 0.921423197 |
| MAP3K7 | 0.918281138 |
| F10 | 0.858001709 |
| F3 | 0.858001709 |
| ADAMTSL1 | 0.858001709 |
| SERPINB1 | 0.858001709 |
| MIR181A1 | 0.858001709 |
| MAP3K1 | 0.838916838 |
| CTBP1 | 0.838916838 |
| CEACAM4 | 0.80468148 |
| PXN | 0.792410553 |
| MALAT1 | 0.78862381 |
| IKBKG | 0.737589896 |
| TFDP1 | 0.737589896 |
| CRYBA1 | 0.737589896 |
| SERPINE1 | 0.731193423 |
| FOXO3 | 0.729933083 |
| ACTG1 | 0.711148858 |
| ARHGDIA | 0.711148858 |
| EZR | 0.711148858 |
| SLC39A6 | 0.711148858 |
| BIN1 | 0.701339006 |
| TIAM1 | 0.701339006 |
| PDPK1 | 0.696958065 |
| SMAD7 | 0.674070597 |
| NTRK3 | 0.64390862 |
| RHOC | 0.64390862 |
| CASP2 | 0.636003375 |
| TNC | 0.612663269 |
| IRF6 | 0.612663269 |
| HOTAIR | 0.609673321 |
| GNE | 0.587587357 |
| XAF1 | 0.587587357 |
| SFRP1 | 0.583380818 |
| MAP2K2 | 0.569063902 |
| CSK | 0.569063902 |
| PIK3C2B | 0.569063902 |
| FOXC2 | 0.569063902 |
| TAGLN | 0.569063902 |
| ARHGDIB | 0.569063902 |
| ENDOG | 0.569063902 |
| FBLIM1 | 0.569063902 |
| CCDC80 | 0.569063902 |
| RACK1 | 0.569063902 |
| PRKD1 | 0.560318828 |
| LDHA | 0.53015697 |
| ANXA2 | 0.53015697 |
| SMARCE1 | 0.53015697 |
| SPP1 | 0.53015697 |
| QSOX1 | 0.53015697 |
| RBFOX2 | 0.53015697 |
| RPS6KA3 | 0.495921612 |
| CDC42 | 0.495921612 |
| MAOA | 0.495921612 |
| PIP5K1C | 0.495921612 |
| JUP | 0.495921612 |
| ATF2 | 0.495921612 |
| NKX2-1 | 0.495921612 |
| NDRG1 | 0.495921612 |
| CRABP2 | 0.495921612 |
| ID2 | 0.495921612 |
| OCLN | 0.495921612 |
| CEACAM8 | 0.495921612 |
| PITPNC1 | 0.495921612 |
| AFAP1L1 | 0.495921612 |
| INSR | 0.455312163 |
| HSPB1 | 0.455312163 |
| PCNA | 0.455312163 |
| GSK3B | 0.455312163 |
| NGF | 0.455312163 |
| TP63 | 0.455312163 |
| CTNNA1 | 0.455312163 |
| KRT14 | 0.455312163 |
| SPHK1 | 0.455312163 |
| EHMT2 | 0.455312163 |
| OGT | 0.455312163 |
| RAC3 | 0.455312163 |
| SIRT6 | 0.455312163 |
| ACP1 | 0.455312163 |
| STK38 | 0.455312163 |
| FOXA1 | 0.455312163 |
| MUC4 | 0.455312163 |
| RHOQ | 0.455312163 |
| ONECUT1 | 0.455312163 |
| S100A7 | 0.455312163 |
| GKN1 | 0.455312163 |
| SRSF3 | 0.455312163 |
| MIR107 | 0.455312163 |
| MIR630 | 0.455312163 |
| LCK | 0.40238893 |
| CDK2 | 0.40238893 |
| DNMT1 | 0.40238893 |
| MERTK | 0.40238893 |
| UCHL1 | 0.40238893 |
| BRCA1 | 0.40238893 |
| MMP3 | 0.40238893 |
| ACTB | 0.40238893 |
| NOS2 | 0.40238893 |
| SLC2A2 | 0.40238893 |
| USP9X | 0.40238893 |
| FYN | 0.40238893 |
| ROR1 | 0.40238893 |
| HSPA1A | 0.40238893 |
| HTRA2 | 0.40238893 |
| SKI | 0.40238893 |
| SPTA1 | 0.40238893 |
| PRDM1 | 0.40238893 |
| TPP2 | 0.40238893 |
| C5AR1 | 0.40238893 |
| CENPF | 0.40238893 |
| LATS2 | 0.40238893 |
| SNAI1 | 0.40238893 |
| TJP1 | 0.40238893 |
| XRCC5 | 0.40238893 |
| CDX2 | 0.40238893 |
| CLDN18 | 0.40238893 |
| DOK2 | 0.40238893 |
| IKZF3 | 0.40238893 |
| S100A11 | 0.40238893 |
| SERPINB5 | 0.40238893 |
| THY1 | 0.40238893 |
| USP11 | 0.40238893 |
| HOXA10 | 0.40238893 |
| LGALS8 | 0.40238893 |
| SNCG | 0.40238893 |
| HTRA3 | 0.40238893 |
| SLPI | 0.40238893 |
| SRPX2 | 0.40238893 |
| IRX1 | 0.40238893 |
| CXCL14 | 0.40238893 |
| EFHD2 | 0.40238893 |
| KIF18A | 0.40238893 |
| ZG16B | 0.40238893 |
| SBSN | 0.40238893 |
| MIR223 | 0.40238893 |
| MIR99A | 0.40238893 |
| MIR451A | 0.40238893 |
| MIR503 | 0.40238893 |
| MIR7-1 | 0.40238893 |
| SNORA80E | 0.40238893 |
